# BECLIN-1/BECN1 at the barrier: a gatekeeper of epithelial and endothelial homeostasis

**DOI:** 10.1080/27694127.2025.2566129

**Published:** 2025-10-05

**Authors:** Juliani Juliani, Walter D. Fairlie, Erinna F. Lee

**Affiliations:** a Cell Death and Survival Laboratory, Olivia Newton-John Cancer Research Institute, Heidelberg, VIC, Australia; b School of Cancer Medicine, La Trobe University, Bundoora, VIC, Australia; c Department of Biochemistry and Chemistry, School of Agriculture, Biomedicine and Environment, La Trobe University, Bundoora, VIC, Australia; d La Trobe Institute for Molecular Science, La Trobe University, Bundoora, VIC, Australia

**Keywords:** Adherens junction, autophagy, tissue barrier, BECLIN-1/BECN1, endocytic trafficking, epidermis, intestinal epithelium, tight junction, epithelium, endothelium

## Abstract

Epithelial and endothelial barriers are essential for tissue homeostasis, protecting the body from environmental insults while regulating selective transport. The integrity of these barriers relies on dynamic intercellular junctions whose composition and organization are constantly remodeled in response to stress and physiological cues. Autophagy and endocytic trafficking are key intracellular pathways that maintain junctional stability and barrier resilience. BECLIN-1 (BECN1), a central regulator of both pathways, coordinates localized membrane dynamics through its interaction with the class III phosphatidylinositol 3-kinase (PtdIns3K) PIK3C3/VPS34. Recent advances reveal that BECN1’s dual role in autophagy and endocytic trafficking is crucial for maintaining barriers in diverse tissues, including the gut, skin, and blood–brain barrier. Conversely, BECN1 dysfunction can compromise junctional integrity, driving inflammatory and degenerative diseases. This review summarizes the emerging evidence linking BECN1 to membrane trafficking, stress adaptation, and immune regulation across barrier tissues, highlighting its potential as a therapeutic target for barrier-associated diseases.

## Introduction

### Background and significance: barrier tissues, junctional proteins and their health implications

Barrier tissues are essential for preserving physiological homeostasis by establishing compartmental boundaries between the internal milieu and the external environment. These tissues are primarily composed of epithelial or endothelial cells that form tightly regulated interfaces with diverse organ-specific functions, such as nutrient absorption in the gut, immune surveillance in the skin and mucosa, gas exchange in the lungs, and solute filtration in the kidney.^[1,2]^ Despite their structural diversity, these barriers share conserved mechanisms that coordinate selective permeability, tissue compartmentalization, and defense against physical, microbial, and inflammatory insults.^[3]^

Epithelial barriers can be single-layered such as in the intestine and alveoli,^[4–6]^ or stratified like the skin and oral mucosa.^[7,8]^ Each is organized to meet distinct physiological demands, for example, the intestinal epithelium balances nutrient uptake while maintaining a critical separation between the gut microbiota and systemic tissues,^[6,9–11]^ whereas the epidermis provides a multi-layered defense against environmental toxins and pathogens.^[12–14]^ In contrast, endothelial barriers, regulate solute and immune cell exchange at vascular interfaces.^[15,16]^ Specialized examples include the blood–brain barrier (BBB), which protects the central nervous system from circulating toxins, and the gut–vascular barrier (GVB), which complements the intestinal epithelium to prevent microbial translocation while permitting nutrient transfer.^[15–19]^

A defining feature across all barrier types is the presence of intercellular junctional complexes, comprising the tight junctions (TJs), adherens junctions (AJs), and in some cases, desmosomes and gap junctions, that regulate paracellular permeability, maintain apicobasal polarity, and provide mechanical stability.^[3,20–28]^ These structures are anchored to the actomyosin cytoskeleton and integrate extracellular signals, allowing rapid modulation of barrier function in response to stress, damage, or inflammation^[29–31]^. In epithelial tissues, junctional complexes confer a high degree of plasticity, enabling dynamic remodeling during normal tissue morphogenesis or in response to injury^[6,29,30,32]^. Endothelial barriers, though typically less plastic, also undergo regulated remodeling during processes such as angiogenesis, immune cell transmigration or vascular leakage during inflammation^[31]^. This capacity for junctional adaptation is critical for maintaining resilience and functional integrity across different barrier types.

Disruption of these junctional structures underlies the pathogenesis of a wide spectrum of diseases. In the gut, barrier dysfunction is implicated in inflammatory bowel disease (IBD), celiac disease, metabolic disorders, and systemic inflammation^[33–36]^. Compromised skin integrity contributes to atopic dermatitis and psoriasis^[37–39]^, while epithelial defects in the lung are associated with asthma, chronic obstructive pulmonary disease, and fibrosis^[40,41]^. Similarly, BBB breakdown facilitates neuroinflammation and neurodegeneration, contributing to diseases such as multiple sclerosis, Alzheimer’s disease, and stroke^[42–44]^. These examples underscore the critical importance of junctional integrity for organ function and the widespread impact of barrier failure in disease.

### Autophagy and endocytic trafficking: key intracellular processes supporting barrier function

Maintaining junctional composition and barrier stability relies on tightly coordinated intracellular processes, notably endocytic trafficking and autophagy, which together regulate the turnover, recycling, and degradation of junctional proteins. These mechanisms allow intercellular junctions to be remodeled in response to developmental cues, environmental challenges, or cellular stress while preserving overall barrier integrity. Endocytic trafficking directs the internalization, sorting, and either recycling or lysosomal degradation of membrane proteins, while autophagy complements this by degrading intracellular components to maintain cellular homeostasis under stress. These pathways will be discussed in greater detail below (see Section “Autophagy and endocytic trafficking in junctional regulation across barrier tissues”).

These fundamental processes are tightly integrated with cytoskeletal dynamics and cell polarity networks that coordinate where and when junctional components are delivered or removed. Disruption at any level can compromise barrier function, resulting in increased permeability, loss of tissue organization, and heightened susceptibility to inflammation or infection.

### BECN1: a central integrator of autophagy and trafficking

While multiple factors regulate barrier maintenance, emerging evidence highlights BECN1 as a key integrator that coordinates both autophagy and endocytic trafficking pathways. Originally identified as a core autophagy protein, BECN1 also regulates endosomal sorting, cargo recycling, cytoskeletal remodeling, and cell death, positioning it at the crossroads of processes essential for epithelial and endothelial barrier function^[45–52]^. Dysfunction in BECN1 disrupts this integration, contributing to barrier breakdown, inflammatory signaling, and disease progression^[47–50,53]^.

In the following sections, we outline the structural and molecular features of epithelial and endothelial barriers, describe how BECN1 orchestrates canonical and non-canonical pathways to maintain barrier homeostasis, and discuss how its dysfunction contributes to disease across diverse organ systems, offering new opportunities for targeted therapies to protect barrier integrity.

## General features and molecular composition of epithelial and endothelial barriers

### Tight junctions: seals of selective permeability

**Figure 1.**
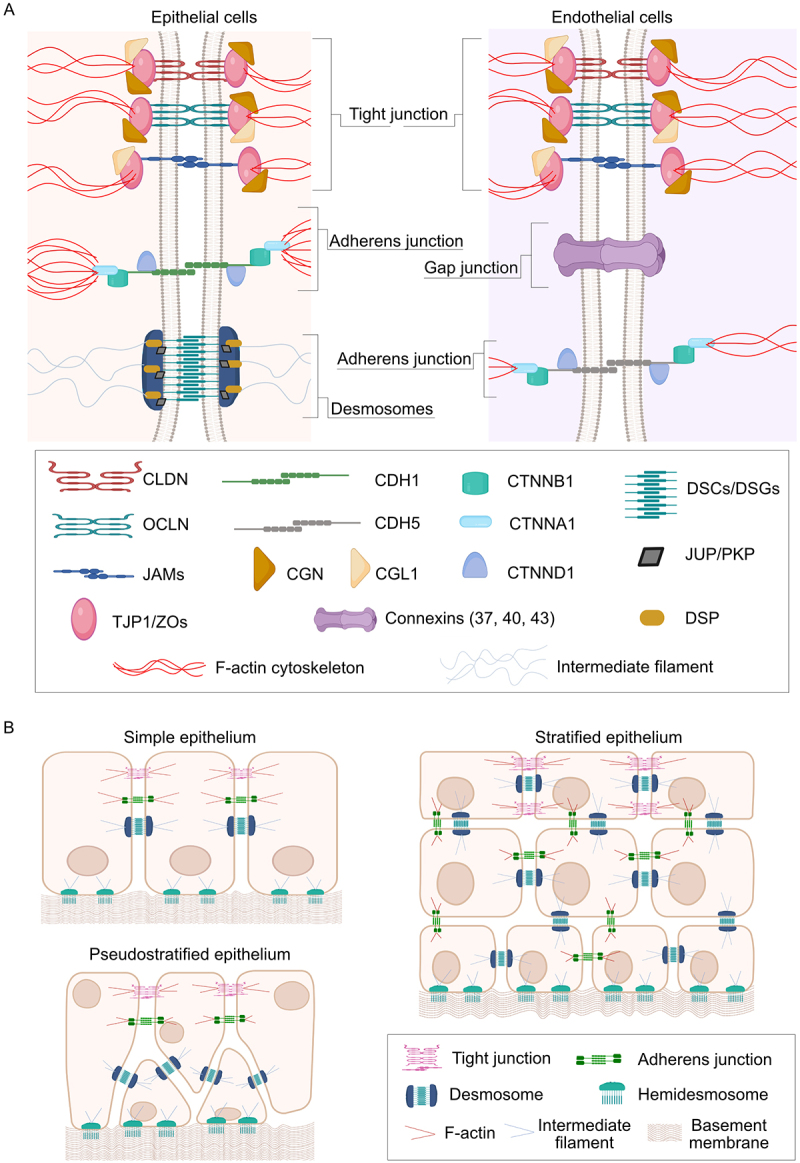
Schematic overview of junctional complexes in epithelial and endothelial cells.

As described in the Introduction, a hallmark of all barrier tissues is their reliance on specialized, multi-protein intercellular junctions (Figure 1A). Tight junctions (TJs) consist of transmembrane proteins including OCLN (occludin), CLDNs (claudins), and JAMs (junctional adhesion molecules), which form selective paracellular seals^[16,27,54,55]^. These proteins are anchored intracellularly by cytosolic scaffold proteins such as TJP1/ZO-1 (tight junction protein 1), MARVELD2/tricellulin (MARVEL domain containing 2), CGN (cingulin), and CGL1/paracingulin (cingulin like 1), which link TJs to the actomyosin cytoskeleton and coordinate junctional signaling^[27,54,56–58]^ (Figure 1A). The composition of CLDN isoforms determines tissue-specific barrier properties. For example, CLDN5 (claudin 5) supports tight paracellular restriction in the BBB,^[59]^ while CLDN2 (claudin 2) permits cation flux in leaky epithelia such as the proximal nephron and small intestine.^[58,60,61]^

(A) Epithelial *versus* endothelial junctions. Epithelial and endothelial barriers rely on multi-protein complexes that regulate paracellular permeability and maintain apicobasal polarity. Tight junctions (TJs) consist of claudins (CLDNs), occludin (OCLN), and junctional adhesion molecules (JAMs), anchored by cytoplasmic scaffolds such as ZO proteins (TJP1) and cingulin (CGN/CGL1). Adherens junctions (AJs) are built from cadherins (CDH1 in epithelia, CDH5/VE-cadherin in endothelia) linked to catenins (CTNNA1, CTNNB1, CTNND1), which tether the complex to the actin cytoskeleton. Desmosomes provide mechanical stability through desmogleins (DSGs) and desmocollins (DSCs) connected *via* plakoglobin (JUP) and plakophilins (PKPs) to intermediate filaments, while gap junctions (connexins) mediate direct intercellular communication. (B) Tissue-specific junctional organization. In simple epithelia, junctions are arranged in a conserved apical-to-basal order (TJ > AJ > desmosomes). Pseudostratified epithelia maintain this arrangement at the apical domain, with desmosomes enriched in suprabasal layers. Stratified epithelia restrict TJs to the upper layers, with desmosomes distributed throughout to ensure tensile strength. Endothelial junctions intermix TJs and AJs along the lateral membrane rather than in strict vertical order. This organizational diversity underpins tissue-specific permeability and mechanical properties. Image created in BioRender.com. Figure 1B adapted from^[3]^. Abbreviations: CDH1, cadherin 1; CDH5, cadherin 5; CGL1, cingulin-like 1; CGN, cingulin; CLDN, claudins; CTNNA1, α-catenin; CTNNB1, β-catenin; CTNND1, p120-catenin; DSC, desmocollins; DSG, desmogleins; JAM, junctional adhesion molecule; JUP, junction plakoglobin; OCLN, occludin; TJP1/ZO, tight junction protein 1; PKP, plakophilin.

### Adherens junctions: anchors for cohesion and polarity

Adherens junctions (AJs), composed of cadherin – catenin complexes, connect adjacent cells *via* calcium-dependent homophilic interactions.^[62]^ Epithelial cells primarily use CDH1/E-cadherin (cadherin 1), which binds intracellular catenins (CTNND1/p120 [catenin delta 1], CTNNB1/beta-catenin [catenin beta 1], and CTNNA1/alpha-E-catenin [catenin alpha 1]) to anchor the complex to cortical actin and coordinate cytoskeletal tension and polarity^[16,23–25,63–66]^ (Figure 1A). In endothelial cells, CDH5/VE-cadherin (cadherin 5) plays a similar role, stabilizing junctions and regulating vascular permeability^[16,67]^ (Figure 1A).

### Desmosomes and gap junctions: strength and communication

Desmosomes, which contribute to mechanical strength, are found in epithelial cells, whereas endothelial cells form gap junctions that mediate intercellular communication^[23,27,68–70]^ (Figure 1A). Despite differences in composition, these junctional structures share common functions in adhesion and barrier maintenance. Desmosomes contain two cadherin families, DSG1-4 (desmoglein 1–4) and DSC1-3 (desmocollin 1–3),^[71]^ whose cytoplasmic domains interact with JUP (junctional plakoglobin) and PKP1-3 (plakophilin 1–3), anchoring the complex to intermediate filaments and conferring mechanical stability.^[72]^ In contrast, gap junctions are formed by the alignment of connexins from adjacent cells, each composed of six subunits (Figure 1A). The human genome encodes 21 connexin genes, while 20 are present in mice, enabling diverse channel properties that support exchange of ions and small signaling molecules between cells.^[73]^

### Junctional organization

Junctional organization varies by tissue architecture and mechanical demands. In simple epithelia (e.g. intestine, bladder), junctions follow a conserved apical-to-basal distribution (TJs > AJs > desmosomes) (Figure 1B), enabling precise control over permeability and transport.^[3]^ In pseudostratified epithelia (e.g. airway mucosa), TJs and AJs seal the apical domain, while desmosomes are enriched in the underlying suprabasal layers, providing mechanical reinforcement^[69,74]^ (Figure 1B). In stratified epithelia (e.g. epidermis), TJs are only restricted to the uppermost layers, whereas desmosomes are distributed throughout the suprabasal layers to maintain cohesion and resist tensile forces^[20,23,68,75]^ (Figure 1B). In contrast, endothelial junctions lack strict apical-basal organization, with TJs and AJs intermingling along the lateral membranes.^[76]^

### Adaptive junctional dynamics and barrier plasticity

Barrier tissues must remain functional under continuously changing physiological conditions. In epithelia, renewal and morphogenetic movements drive ongoing junctional remodeling.^[66,77–81]^ Endothelial cells experience hemodynamic forces, including shear stress and cyclic stretch, which necessitate dynamic cytoskeletal and junctional adaptation.^[82,83]^ This is achieved by modulating junctional protein expression, composition, spatial organization, and localization in real-time.^[67,82,84–86]^

The functional impact of such modulation is evident across different tissues. For example, the BBB exhibits high transendothelial electrical resistance due to densely packed TJ networks^[85,87–89]^, whereas mesenteric capillaries and postcapillary venules display more loosely organized junctions, allowing greater paracellular permeability.^[70,90]^ Similarly, the GVB exhibits a permissive junctional architecture aligned with nutrient absorption.^[19]^

Experimental models demonstrate how junctional protein regulation impacts barrier function. For example, CLDN18 (claudin 18) deficiency increases alveolar epithelial permeability and reduces transepithelial resistance^[91]^, while mechanical stretch upregulates OCLN and ZO-1 at endothelial junctions.^[92]^ In intestinal models, OCLN downregulation similarly increases paracellular permeability.^[93]^ During morphogenetic processes, CDH5 redistribution supports endothelial motility^[94]^ and CDH1 endocytosis governs epithelial tissue architecture and rearrangement.^[95,96]^ These examples underscore how adaptive junctional dynamics underpin barrier function across diverse tissues.

## Autophagy and endocytic trafficking in junctional regulation across barrier tissues

### Overview of the role of endocytic trafficking and autophagy in junctional turnover

Maintaining junctional protein composition, localization, and stability in both epithelial and endothelial barriers is essential for preserving tissue integrity and function. While transcriptional programs establish baseline expression of junctional components, rapid and precise post-translational remodeling enables these barriers to respond dynamically to physiological and pathological cues. Notably, junctional reorganization can occur within an hour, significantly faster than the typical 6- to 12-hour half-lives of key junctional proteins, highlighting the importance of fast-acting regulatory mechanisms.^[97–99]^

As outlined in the Introduction , endocytic trafficking and autophagy are central to these rapid adjustments. Endocytic trafficking governs the internalization, sorting, recycling, or degradation of junctional proteins, ensuring precise spatial and temporal control of their surface availability.^[3,100–105]^ In parallel, basal autophagy complements this process by degrading damaged or surplus components and supporting cell viability, especially under conditions of metabolic stress.^[106–108]^ In addition to these vesicle-based pathways, lateral membrane diffusion enables redistribution of junctional proteins within the plasma membrane itself, providing a rapid mechanism for local junctional remodeling without requiring vesicular transport.^[109,110]^ Together, the interplay of these membrane-intensive pathways facilitates timely junctional remodeling, allowing barriers to remain resilient and adapt to fluctuating environmental or inflammatory stressors.

### Junctional remodeling mechanisms under homeostatic conditions

CDH1/E-cadherin at AJs provides a representative example of how membrane trafficking pathways coordinate to maintain continuous junctional turnover under steady-state.^[103,111]^ Internalized CDH1 can undergo RAB4A (RAB4A, member RAS oncogene family)/RAB11A (RAB11A, member RAS oncogene family)-mediated trafficking, allowing direct recycling to the apicolateral membrane or relocation from lateral regions to the apical domain *via* targeted endocytosis and transport.^[109,112–114]^ Alternatively, CDH1 can be repositioned within the plasma membrane by lateral diffusion, allowing relocalization to apical junctional domains without vesicular involvement.^[109,114,115]^ This dynamic redistribution maintains junctional integrity while fine tuning remodeling during epithelial tissue maintenance and morphogenesis.

Tight junction (TJ) components follow similar principles of regulation. OCLN continuously cycles between the plasma membrane and intracellular compartments, with a portion routed back to the membrane *via* recycling endosomes and the remainder targeted for lysosomal degradation which is balanced by biosynthetic replenishment.^[116]^ CLDNs are subject to post-translational regulation, including ubiquitination, which targets them for internalization and sorting through the endosomal system. Once internalized, they engage the endosomal sorted complexes required for transport (ESCRT) machinery, which directs them either for recycling back to the plasma membrane or for lysosomal degradation, depending on the cellular context and modification state.^[117–119]^ Notably, some CLDNs are sequestered in double-membraned vesicles resembling autophagosomes, formed by engulfment of junctional membrane from adjacent cells. These vesicles undergo lysosomal fusion and degradation, highlighting a role for autophagy in junctional CLDNs turnover alongside classical endocytic routes.^[120]^

Gap junctions and desmosomes turnover are also tightly regulated. Connexins, the core components of gap junctions, have high turnover rates across many tissue types.^[121,122]^ They are internalized *via* clathrin-mediated endocytosis and sorted for degradation through multiple pathways, including classical RAB5A/RAB5 (RAB5A, member RAS oncogene family):RAB7A/RAB7 (RAB7A, member RAS oncogene family)-mediated endolysosomal trafficking and direct lysosomal fusion of double-membraned connexosomes formed during gap junction internalization.^[123–127]^ In some contexts, connexins can be recycled back to the plasma membrane *via* RAB11A^+^ vesicles and reassembled into functional junctions or degraded through autophagy under cytosolic stress conditions.^[128–130]^ In contrast, desmosomal remodeling mechanisms have only recently begun to be elucidated. For example, DSG1 (desmoglein 1) has been shown to interact with the retromer component VPS35 (VPS35 retromer complex component) to facilitate retrograde trafficking and plasma membrane recycling, supporting keratinocyte differentiation and epithelial stratification.^[131]^ Collectively, these mechanisms sustain junctional homeostasis while allowing for controlled remodeling during tissue maintenance, growth and morphogenesis. Their dynamic nature ensures that barrier function remains robust yet adaptable to subtle physiological fluctuations. However, these pathways are equally vital for supporting junctional adaptation under stress, when rapid reorganization determines barrier resilience or breakdown.

### Junctional remodeling mechanisms in response to external cues

Beyond homeostatic regulation, endocytic trafficking and autophagy play critical roles in facilitating junctional remodeling in response to external stimuli. In epithelial cells, perturbations such as calcium depletion, actin depolymerization, and mechanical stress induce the internalization of TJ components, including OCLN, CLDNs, and ZO proteins, *via* clathrin-mediated or independent endocytic mechanisms.^[100]^ Internalized proteins are subsequently sorted through the endosomal system for either recycling or lysosomal degradation, allowing the epithelial barrier to adapt structurally to environmental conditions.^[132–134]^ In endothelia, VEGFA (vascular endothelial growth factor A, an angiogenic factor) stimulation triggers OCLN phosphorylation at Ser490, leading to its ubiquitination and endolysosomal degradation, which contributes to increased vascular permeability.^[135]^

Adherens junctions (AJ) also undergo rapid remodeling following stress. Under conditions such as calcium depletion or epithelial injury, CDH1 is rapidly internalized and either recycled to restore junctions or degraded to facilitate tissue remodeling.^[101,109,133,136]^ Post-translational modifications further influence AJ dynamics; for example, phosphorylation of CTNNB1 at Tyr654 reduces its binding affinity for CDH1, promoting dissociation and internalization of the AJ complex.^[137]^ These changes are critical for physiological processes such as epithelial–mesenchymal transition (EMT), wound repair, and developmental morphogenesis.^[138–142]^

Environmental stressors and inflammatory signals also promote junctional remodeling, which can be disruptive. Cytokines such as TNF/TNF-alpha (tumor necrosis factor) and CCL2 (C-C motif chemokine ligand 2),^[143,144]^ infection,^[145,146]^ and metabolic stress induce the endocytosis of TJ proteins including CLDNs, OCLN, JAM-A, and ZO-1, driving barrier disruption and increased permeability.^[100,147]^ At the BBB, methamphetamine and VEGF similarly trigger OCLN internalization, contributing to heightened paracellular flux.^[135,144,148]^

Autophagy intersects with these stress-induced processes by selectively degrading junctional proteins. For example, in intestinal epithelial cells (IECs), starvation-induced autophagy targets CLDN2 for degradation leading to decreased permeability along the paracellular pathway and enhancing TJ barrier function.^[149]^ In porcine models, amino acid deprivation leads to downregulation of CLDN1 and ZO-1 compromising junctional integrity, but this is rescued by activating autophagy with rapamycin.^[150]^ In ischemia-reperfusion models, autophagy induction mitigates ZO-1 loss and preserves BBB integrity.^[151]^ Nitric oxide generated during ischemic injury directs CLDN5 to autophagosomes for lysosomal degradation *via* CAV1 (caveolin 1)-mediated endocytosis,^[152]^ reinforcing the integration of autophagy and endocytic machinery in stress-responsive junctional turnover.

Together, these findings underscore the role of endocytic trafficking and autophagy in dynamic junctional regulation, maintaining barrier resilience while supporting necessary structural adaptations. Notably, BECN1 has emerged as a key integrator of both these pathways, coordinating autophagy and endosomal trafficking to modulate junctional organization and barrier integrity across diverse tissues.

## BECN1 structure and its interactome

### Discovery and tumor suppressor function

BECN1 was the first mammalian autophagy regulator to be identified, originally discovered as the orthologue of the yeast *Apg6/Vps30* gene. This discovery stemmed from a yeast two-hybrid screen for proteins interacting with the anti-apoptotic protein BCL2 (BCL2 apoptosis regulator).^[153]^ Shortly after, genomic studies revealed *BECN1* as a candidate tumor suppressor, with the *BECN1* gene mapped to the tumor-susceptibility locus 17q21, monoallelically deleted in 40–75% of sporadic breast, ovarian and prostate cancers.^[154]^ This tumor-suppressive role was substantiated in murine models, where heterozygous *Becn1* deletion led to high incidences of spontaneous tumorigenesis, establishing it as a haploinsufficient tumor suppressor gene.^[155,156]^

### Structural domains and complex formation

**Figure 2.**
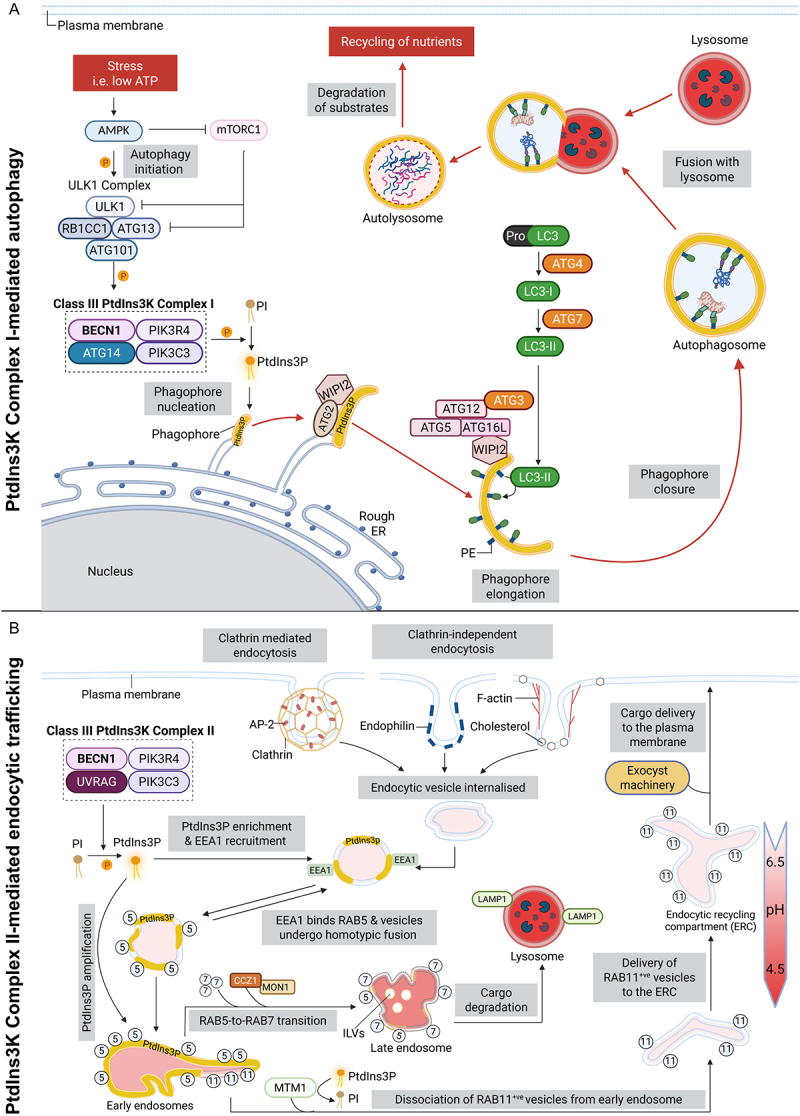
Schematic representation of BECN1 coordinating autophagy and endocytic trafficking through distinct class III PtdIns3K complexes.

BECN1 contains three conserved structured domains: a BH3 (BCL2 homology domain 3) domain, coiled-coil domain (CCD) and evolutionary conserved domain (ECD).^[46]^ The BH3 domain is preceded by a long (~150 amino acid) unstructured N-terminal domain.^[157]^ These domains provide multiple sites for post-translational modifications and binding sites for various BECN1-interacting proteins that can positively or negatively modulate BECN1’s activity and facilitate its diverse protein interactome.^[46,158]^ Through these interactions, BECN1 serves as a core scaffold for assembling two functionally distinct class III PtdIns3K complexes. Complex I consists of BECN1, PIK3C3/Vps34 (phosphatidylinositol 3-kinase catalytic subunit type 3), PIK3R4/VPS15 (phosphoinositide-3-kinase regulatory subunit 4), and ATG14/ATG14L (autophagy-related 14) and initiates autophagy (Figure 2A). In Complex II, ATG14L is replaced by UVRAG (UV radiation resistance associated), and regulates endocytic trafficking^[46]^ (Figure 2B).

(A) Class III PtdIns3K Complex I-mediated autophagy. Stress conditions (e.g. nutrient/energy depletion) activate AMPK and ULK1, while suppressing mTORC1, promoting assembly of the BECN1–VPS34/PIK3C3–VPS15/PIK3R4–ATG14 Complex I. This generates PtdIns3P on nascent phagophore membranes, recruiting WIPI2 and ATG2 to tether donor membranes and drive lipid transfer for expansion. WIPI2 also recruits the ATG12–ATG5–ATG16L1 complex, which together with ATG7/ATG3, mediates LC3 lipidation, enabling phagophore elongation and closure. Mature autophagosomes then fuse with lysosomes to form autolysosomes where substrates are degraded and recycled. (B) Class III PtdIns3K Complex II-mediated endocytic trafficking. Internalized cargo enters early endosomes *via* clathrin-dependent or -independent pathways. The BECN1–VPS34/PIK3C3–VPS15/PIK3R4–UVRAG Complex II produces PtdIns3P, defining early endosome identity and recruiting EEA1 to tether and fuse vesicles. The CCZ1–MON1 complex mediates RAB5-to-RAB7 conversion, driving maturation into late endosomes. Cargo is either targeted to lysosomes for degradation or diverted into recycling routes: RAB11A^+^ vesicles are released from early endosomes by MTM1 activity and delivered to the endocytic recycling compartment before exocyst-dependent return to the plasma membrane. The red gradient arrow represents progressive acidification (pH ~ 6.5 to 4.5). Circled numbers correspond to RAB5A, RAB7A, and RAB11A. BECN1-containing complexes are shown within dotted line boxes. Image created in BioRender.com. Abbreviations: AMPK, 5' adenosine monophosphate-activated protein kinase; AP-2, adaptor protein 2; ATG, autophagy-related; mTORC1, mechanistic target of rapamycin complex 1; MTM1, myotubularin 1; RB1CC1/FIP200, RB1 inducible coiled-coil 1; ULK1, unc-51-like autophagy activating kinase 1; WIPI2, WD repeat domain, phosphoinositide interacting 2.

### BECN1 interactome and regulation

BECN1’s activity can also be modulated by binding to various interacting partners. For example, it can bind to BCL2 family members such as BCL2 and BCL2L1/BCL-XL (BCL2 like 1), *via* its BH3 domain, leading to its sequestration and functional inhibition.^[159,160]^ Under cellular stress conditions, such as nutrient deprivation, this interaction is disrupted through mechanisms including phosphorylation of either BECN1 or BCL2, freeing BECN1 to initiate autophagy.^[161,162]^ BECN1-binding proteins, such as AMBRA1 (autophagy and beclin 1 regulator 1), a protein essential for nervous system development,^[163]^ and HMGB1 (high mobility group box 1), a non-histone DNA binding protein,^[164]^ can also competitively displace BCL2, stabilizing BECN1 complexes and enhancing its autophagic functions.^[165,166]^ Additionally, binding of NBRF2 (nuclear receptor binding factor 2) to ATG14L in class III PtdIns3K Complex I has been shown to promote autophagy,^[167,168]^ although one study suggests that this subcomplex inhibits autophagy.^[169]^ Alternatively, binding of RUBCN (rubicon autophagy regulator) to UVRAG in class III PtdIns3K Complex II negatively regulates BECN1 activity, reducing its autophagy function at the maturation stage as well as endocytic trafficking.^[170]^

Beyond protein–protein interactions, BECN1 is subject to multi-layered regulation at the transcriptional, post-transcriptional, and post-translational levels. Transcription factors such as E2F1 (E2F transcription factor 1) and NF-kappaB upregulate *Becn1* expression, while epigenetic repression and microRNAs (e.g., miR-30a, miR-376b) can downregulate its transcription.^[171–176]^ At the protein level, BECN1 undergoes various post-translational modifications, such as phosphorylation, ubiquitination, ISGylation, acetylation and proteolytic cleavage.^[46]^ These modifications dynamically alter BECN1 functionality by inducing conformational changes that affect BECN1’s binding affinity or activate/inactivate it for interactions with autophagy-related protein partners.^[46,158]^ For instance, phosphorylation by kinases such as ULK1 (unc-51 like autophagy activating kinase 1) and 5’ adenosine monophosphate-activated protein kinase (AMPK) under amino-acid-depleted conditions activates autophagy,^[177,178]^ whereas phosphorylation by AKT/protein kinase B (AKT serine/threonine kinase) and BCR (breakpoint cluster region)-ABL1 (ABL proto-oncogene 1, non-receptor tyrosine kinase) inhibits autophagy.^[179,180]^

This multifaceted regulation underscores BECN1’s central role in cellular homeostasis. Acting as a signal integration node, BECN1 orchestrates the production of phosphatidylinositol 3-phosphate (PtdIns3P), a critical lipid for membrane dynamics in both autophagy and endocytic trafficking, processes essential for junctional remodeling and barrier maintenance. Understanding how PtdIns3P distribution supports these distinct, yet interconnected pathways provides insight into BECN1’s coordinating function in these processes.

## BECN1-mediated PtdIns3P production coordinates autophagy and endocytic trafficking

### General features of autophagosome and endosome membranes

Both autophagy and endocytic trafficking are membrane-intensive pathways that enable dynamic cargo turnover and stress adaptation, as outlined in earlier sections. Autophagosomes, the hallmark structures of autophagy, are double-membraned vesicles that sequester cytoplasmic material for delivery to lysosomes, culminating in autolysosome formation.^[181]^ By contrast, endocytic trafficking is mediated by a highly dynamic network of single-membraned tubulo-vesicular endosomes that sort, recycle, or degrade extracellular cargoes and membrane proteins to maintain surface composition and polarity.^[182]^

Critically, both systems rely on the localized generation of PtdIns3P on specific membrane subdomains, which serves as a molecular signal to recruit effectors that orchestrate vesicle formation, cargo sorting, and membrane remodeling.^[181,183]^ This PtdIns3P landscape is shaped by the class III PtdIns3K PIK3C3/Vps34 in complex with BECN1, which acts as a scaffold integrating upstream signals to direct PtdIns3P production for both autophagy and endocytic pathways.^[46,182,184,185]^ This dual control underscores how BECN1 sits at the interface of these vesicular networks, linking membrane dynamics to barrier integrity and stress resilience.

### Class III PtdIns3K complex I-mediated autophagy initiation

Autophagy is initiated upon nutrient or energy stress *via* AMPK- and ULK1-mediated phosphorylation of BECN1, which promotes assembly of class III PtdIns3K Complex I, consisting of PIK3C3/Vps34, PIK3R4/VPS15, ATG14, and BECN1.^[45,186–188]^ This process coincides with inhibition of mechanistic target of rapamycin complex 1 (mTORC1), relieving its suppressive effect on the ULK1 complex and further facilitating ULK1 activation.^[107]^ Assembly of class III PtdIns3K Complex I activates the lipid kinase activity of PIK3C3/Vps34, enabling the production of PtdIns3P through phosphorylation of PI found in vesicular membranes destined for autophagosome formation. This leads to the nucleation of a nascent flattened double-membraned structure known as the “phagophore,” marking the initiation of autophagy.^[107,185]^

PtdIns3P recruits effectors such as WIPI1/2 (WD repeat domain, phosphoinositide interacting 1/2) and ATG2A/ATG2B (autophagy-related 2A/autophagy-related 2B), which mediates lipid transfer and membrane expansion to facilitate autophagosome growth.^[189–192]^ WIPI2 also recruits the ATG12 (autophagy-related 12)-ATG5 (autophagy-related 5) – ATG16L1 (autophagy-related 16 like 1) complex, facilitating MAP1LC3B (microtubule associated protein 1 light chain 3 beta) lipidation via ATG7 (autophagy-related 7) and ATG3 (autophagy-related 3), which supports autophagosome elongation and maturation.^[45,181,190,193,194]^ The mature autophagosome subsequently fuses with the lysosome to form an autolysosome, enabling degradation and recycling of intracellular cargo.^[185,195]^ A schematic overview of this process is shown in Figure 2A.

### PtdIns3k complex II-driven endocytic trafficking

Endocytic trafficking mediates the internalization and sorting of macromolecules and membrane proteins *via* single-membraned vesicles.^[182,196]^ Cargoes enter early endosomes through clathrin-mediated or clathrin-independent endocytosis,^[111,197]^ where BECN1 forms class III PtdIns3K Complex II with PIK3C3, PIK3R4, and UVRAG to promote PtdIns3P production.^[46,198–201]^ PtdIns3P recruits EEA1 (early endosome antigen 1), a tethering protein that facilitates early endosome fusion and cargo sorting.^[202]^ It also supports the recruitment of the MON1 (MON1 vesicular trafficking associated)-CCZ1 (CCZ1 vacuolar protein trafficking and biogenesis associated) complex, driving the RAB5-to-RAB7 conversion required for endosome maturation into late endosomes.^[203]^ Furthermore, as the early endosome matures, there is an increase in the formation of intraluminal vesicles as well as the acidification of the endosomal lumen (pH ~ 5.5), which promotes further ligand-receptor dissociation and prepares cargo for lysosomal degradation.^[198,204–206]^

Alternatively, cargo destined for recycling back to the plasma membrane is sorted away from the degradative route at the early endosome and directed into the recycling pathway (Figure 2B). These cargoes only transiently associate with the RAB5^+ve^ sorting endosomes and rely on sequential sorting into the RAB11A^+ve^ peri-nuclear endocytic recycling compartment (ERC), from which they are redirected to the plasma membrane.^[207,208]^ In contrast to RAB7 recruitment onto RAB5^+ve^ endosomes by PtdIns3P during endosomal maturation, inactive RAB11A is already present on peripheral RAB5^+ve^ endosomes and is activated by local generation and enrichment of PtdIns3.^[209]^ Following activation of RAB11A, the phosphatidylinositol 3-phosphatase, MTM1 (myotubularin 1), is recruited to PtdIns3P-enriched domains, where it converts PtdIns3P to PI, leading to rapid dissociation of RAB11A^+ve^ vesicles from the RAB5^+ve^ endosomes.^[210]^ These newly formed RAB11A^+ve^ vesicles are then delivered to the ERC, followed by recruitment of the exocyst machinery, for cargo re-delivery to the plasma membrane.^[210]^ A schematic overview is shown in Figure 2B.

Together, these mechanisms demonstrate how BECN1, by scaffolding the class III PtdIns3K PIK3C3/Vps34, coordinates the generation of localized PtdIns3P pools that integrate autophagy and endocytic trafficking. This dual regulation ensures precise vesicle formation, cargo sorting, and membrane remodeling, processes essential for dynamic junctional organization and barrier homeostasis in epithelial and endothelial tissues. The following sections provide examples of BECN1’s tissue-specific roles in barrier regulation, focusing on the gut and skin epithelia and the blood–brain barrier endothelium (Table 1).

**Table 1.** Experimental Models and Tissue-Specific Functions of BECN1 in Barrier Organs Under Physiological and Pathological Conditions.

	Genetic background/experimental model/samples (species)	Physiological condition(s)/ Injury model(s)	Major phenotypes/altered signaling pathway	References
Intestinal epithelium	*Becn1*^*fl/fl*^ *Villin-Cre* and *Becn1*^*fl/fl*^ *Villin-Cre-*derived intestinal organoids (M*us musculus*)	Homeostasis	Fatal enteritis, villus atrophy, loss of secretory IECs. Abnormal Paneth cell morphology. Stressed ER and mitochondria. Mistrafficking of CDH1 and OCLN. Cargo stalling within RAB5^+ve^ early endosomes Impaired cargo trafficking to the lysosome Disorganized F-actin cytoskeleton and loss of actin belt.	Tran et al.^[49]^ Juliani et al.^[233]^
	C57BL/6 (M*us musculus*)	Treatment with Tat-BECN1	Increased paracellular mannitol flux.	Wong et al.^[48]^
		*Salmonella* Typhimurium infection	Activates BECN1 *via* MAPK8-mediated dissociation from BCL2-BECN1 complex, promoting autophagy-dependent bacterial clearance.	Li et al.^[239]^
	*Becn1*^*F121A*^ (M*us musculus*)	Homeostasis	Reduced ER stress. Enhanced colonic mucus barrier. Enrichment of protective gut microbiota.	Naama et al.^[236]^
		3% Dextran sulfate sodium (5–7 days)	Reduced weight loss, disease severity, colon shortening, and histological damage. Barrier function preserved.	
		Adherent-invasive *Escherichia coli* infection	No chronic intestinal inflammation or colon shortening.	
	CGAS^−/−^ (M*us musculus*)	2% dextran sulfate sodium (7 days)	More severe weight loss, disease severity, colon shortening and tissue damage. Increased inflammatory infiltrate and IEC apoptosis. Thickened lamina propria and muscularis mucosa – all associated with reduced BECN1 levels.	Khan et al.^[237]^
	RAW264.7 macrophages (*Mus musculus*)- shRNA-mediated YTHDC1 knockdown	LPS/IFNγ induced inflammation	Promotes NF-kappaB signaling by destabilizing *Becn1* mRNA and suppressing autophagy.	Zhou et al.^[238]^
	Wistar rat (*Rattus norvegicus)*-following maternal separation during newborn to induce irritable bowel syndrome	Standard diet	Increased intestinal inflammation, oxidative stress and impaired autophagy flux due to reduced BECN1 and MAP1LC3B levels.	Chimienti et al.^[252]^
	*Atg6[∆ISC] (Drosophila melanogaster*)	Homeostasis	ISC hyperproliferation, centrosome amplification, DNA damage accumulation.	Na et al.^[234]^
	*bec-1(ok691)* null and *bec-1*(ok700) hypomorphic mutants (*Caenorhabditis elegans*)	Homeostasis	Embryonic lethal. Defective retrograde transport and retromer function. Mistrafficking of WLS/mig-14 to lysosome. Impaired apoptotic corpse clearance. Intestinal vacuole accumulation.	Ruck et al.^[235]^
	Caco-2 cells (*Homo sapiens*)	BECN1 siRNA-mediated knockdown	Enhanced TJ barrier function.	Wong et al.^[48]^
		Treatment with Tat-BECN1	Increased OCLN endocytosis, reduced total OCLN level. Reduced TJ barrier function.	
	Ulcerative colitis colonic mucosa (H*omo sapiens*)		Significantly higher BECN1 levels compared to control.	Hao et al.^[248]^
	Colorectal cancer patient tissues (H*omo sapiens*)		Higher BECN1 levels associated with longer overall survival, whereas reduced expression is linked to poorer survival.	Li et al.^[249]^ Koukourakis et al.^[246]^
	Pediatric celiac disease intestinal biopsies (H*omo sapiens*)		Reduced *BECN1* expression in active disease.	Comincini et al.^[247]^
Skin epithelium	*Becn1*^*fl/fl*^*-K5-Cre* (*Mus musculus*)	Homeostasis	Neonatal lethality. Stiff and shiny skin, rapid dehydration, excessive trans epidermal water loss, weight loss, increased skin permeability, impaired epidermal stratification and differentiation. Mistrafficking of ITGA6 due to defective early and recycling endosome function.	Noguchi et al.^[50]^
	Sk-Mel-28 and A375 melanoma cell lines (*Homo sapiens*)	Homeostasis	SIRT1-BECN1 axis promotes autophagic degradation of CDH1, driving EMT and invasion.	Sun et al.^[142]^
	Chronic plaque psoriasis (*Homo sapiens*)		Increased BECN1 expression in psoriatic skin.	Amer et al.^[263]^
	Keloid tissues (*Homo sapiens*)		Increased BECN1 expression.	Jeon et al.^[264]^
	Hypertrophic scar tissues (*Homo sapiens*)		Decreased BECN1 levels.	Shi et al.^[267]^
Blood-brain barrier	*Becn1*^*fl/fl*^*-Pcp2-Cre* (*Mus musculus*)	Homeostasis	Accelerated Purkinje cell degeneration, accompanied by ataxia. Mislocalized PtdIns3P, failed endosomal targeting. Impaired EEA1 recruitment; enlarged LAMP1^+ve^ lysosomes.	McKnight et al.^[51]^
	*Becn1*^*fl/fl*^*-EMX-Cre (Mus musculus*)	Homeostasis	Severe hippocampal neurodegeneration with apoptotic neuronal loss.	
	C57BL/6 – TBI induction as described in^[280]^ (*Mus musculus*)	Di-3n-butylphthalide	Improved motor function after TBI, attributed to reduced BECN1 levels, which increased TJs and AJs protein expression. Restored BBB integrity and reduced neuronal death.	Wu et al.^[283]^
	C57BL/6 model of acute ischemic stroke *via* transient middle cerebral artery occlusion (*Mus musculus*)	Homeostasis	Increased BBB permeability, coinciding with increased RUBCN expression.	Lin et al.^[285]^
		ROCK1 treatment	Decreased RUBCN-BECN1 interaction, attenuated BBB disruption and brain injury.	
	bEnd.3 cells (*Mus musculus*)	Oxygen-glucose deprivation	Increased RUBCN expression association with BECN1, impaired autophagy flux and endothelial cell viability.	
	Primary hippocampal and cortical neuron culture – *Becn1* shRNA-mediated knockdown (*Mus musculus*)	Homeostasis	Accelerated neuronal death. Defective RAB11- and retromer-mediated recycling of TGFB1R1.	O’brien et al.^[278]^
	BV2 and N9 microglial cells-*Becn1* shRNA-mediated knockdown (*Mus musculus*)	Homeostasis	Prominent reduction in retromer complex, leading to reduced phagocytic receptor recycling.	Lucin et al.^[262]^
	Male Sprague-Dawley rats – TBI model using Feeney’s weight-drop method (*Rattus norvegicus*)		Elevated BECN1 levels, increased autophagy-mediated degradation of TJ proteins and induce BBB damage.	Bi et al.^[281]^
	C6 astrocytes -*Becn1* shRNA knockdown (*Rattus norvegicus*)	Homeostasis	Impaired phagocytic uptake of latex beads due to reduced retromer levels and impaired recruitment to phagosomes.	Silva et al.^[279]^
	*becn1*^*-/-*^ *(Danio rerio)*	Hypoxia	Severe cytosolic accumulation of CLDN5 in BMEC.	Yang et al.^[275]^
	New Zealand white rabbits (*Oryctolagus cuniculus*)	Induction of ischemic/reperfusion injury	Increased BECN1 levels due to ROS-driven NF-kappaB signaling.	Zeng et al.^[288]^
	HUVECs (*Homo sapiens*)	Lethal ischemia treatment	Suppressed *BECN1* upregulation despite upregulation of other autophagy and lysosomal proteins.	Ma et al.^[286]^
		Sublethal ischemia/reperfusion	Upregulated BECN1 expression, in an NF-kappaB-dependent manner.	Zeng et al.^[287]^
	Microglia from postmortem AD patients (*Homo sapiens*)		Reduced BECN1, associated with reduced retromer complex.	Lucin et al.^[262]^
	CSF from TBI patients (*Homo sapiens*)		Elevated BECN1 levels.	Au et al.^[280]^
	Brain tissues from TBI patients (*Homo sapiens*)		Elevated BECN1 levels.	Clark et al.^[282]^

## BECN1 in the intestinal epithelium

### BECN1 in the intestinal epithelium during normal physiology

The intestinal epithelium serves as a critical barrier separating the gut lumen from the host’s internal environment.^[9]^ It consists of a single layer of specialized, polarized IEC types arranged to perform distinct functions.^[11,65,211]^ As a front-line defense, the intestinal epithelium is continuously exposed to external insults including pathogenic microbes, fluctuating pH, and mechanical stresses from bowel movements. As described in previous sections, its barrier integrity is maintained by intercellular junctional complexes, primarily TJs and AJs, and a dynamic cytoskeletal network composed of actin filaments, intermediate filaments and microtubules.^[34,212–215]^ Together, these components regulate paracellular permeability, preserve epithelial polarity, and maintain tissue architecture.

Autophagy is a critical regulator of intestinal epithelial homeostasis. It supports multiple aspects of epithelial function, including clearance of intracellular pathogens (xenophagy),^[216–219]^ regulation of junctional protein composition,^[149,220]^ and maintenance of specialized IEC types such as Paneth cells, goblet cells, and intestinal stem cells (ISCs).^[107,221–232]^ Disruption of core autophagy genes such as *Atg5*, *Atg7*, or *Atg16l* in mice leads to epithelial dysfunction, altered microbiota composition, and increased susceptibility to intestinal injury and inflammation.^[45,107]^ These broader aspects of autophagy in IEC homeostasis, beyond the specific role of BECN1, have been reviewed elsewhere^[45,107]^ and will not be discussed here in further detail.

However, while autophagy regulators such as ATG5 or ATG7 are important for IEC homeostasis, they are not strictly essential for epithelial survival under basal conditions. In contrast, deletion of *Becn1* in IECs results in rapid and fatal epithelial breakdown, underscoring BECN1’s essential role in maintaining intestinal barrier integrity^[49,233]^ (Table 1). This indispensability stems from BECN1’s dual roles in autophagy and endocytic trafficking, demonstrated in diverse models. For example, in Caco-2 cells, BECN1 regulates permeability by modulating OCLN endocytosis^[48]^ (Table 1). In *D. melanogaster*, loss of the *BECN1* orthologue, *Atg6*, in ISCs induces age-related phenotypes including hyperproliferation, centrosome amplification and DNA damage, compromising ISC function and epithelial barrier maintenance^[234]^ (Table 1). Similarly, knockdown of *Bec-1* in *C. elegans* results in aberrant vacuole accumulation in the intestines, defective retrograde endosome-to-Golgi transport, and impaired clearance of apoptotic corpses^[235]^ (Table 1). Conversely, constitutive BECN1 activation (*Becn1*^*F121A*^ mutant mice) protects against colitis by enhancing goblet cell function through autophagy-mediated alleviation of ER stress^[236]^ (Table 1).

Our recent work in adult mice shows that IEC-specific *Becn1* deletion (using the *Vil1-CreERT2* system) provides direct mechanistic evidence of BECN1’s essential role in endocytic trafficking, cytoskeletal organization, and barrier maintenance.^[49]^ Intestinal organoids derived from these mice recapitulated the epithelial breakdown independent of stromal, immune, or microbial components, confirming an IEC-intrinsic requirement for BECN1.^[49]^ Importantly, these phenotypes were not observed in *Atg5-, Atg7-, Atg14-*, or *Atg16l-*deficient mice, implicating non-redundant BECN1 functions in endocytic trafficking and cytoskeletal regulation.^[49,107,233]^ Mechanistically, BECN1 loss caused cargo stalling within RAB5^+ve^ early endosomes, impairing recycling and degradation of junctional proteins such as CDH1 and OCLN. This led to apical F-actin disruption, loss of epithelial polarity, and compromised barrier integrity^[49,233]^ (Table 1). However, whether endocytic trafficking defects are the sole cause of the striking gut phenotype observed following *Becn1* loss, or are exacerbated by additional loss of autophagy signaling, is unclear. Generation of mice with deficiency in an endocytic trafficking-only regulator, such as *Uvrag*, could help further dissect this important question, though Drosophila models (see below) clearly highlight the importance of Complex II in the gut.

BECN1 also contributes to intestinal barrier homeostasis through regulation of immune responses, either *via* its role in IECs or in immune cells. The DNA sensor CGAS (cycling GMP-AMP synthase) interacts with BECN1 in IECs to promote autophagy and suppress IEC death during inflammation in IBD^[237]^ (Table 1). In colonic macrophages, the RNA-binding protein YTHDC1 (YTH N6-methyladenosine RNA binding protein C1), a regulator of N6-methyladenosine epigenetic modification, stabilizes *Becn1* mRNA to maintain autophagy, restrains NF-kappaB signaling, and its loss increases inflammation in dextran sodium sulfate-induced colitis^[238]^ (Table 1). During *S. typhimurium* infection, BECN1 is also activated *via* MAPK8/JNK1 (mitogen-activated protein kinase 8)-mediated dissociation from the BCL2-BECN1 complex, facilitating autophagy-dependent bacterial clearance^[239]^ (Table 1).

Gut phenotypes associated with loss of BECN1-interacting proteins also underscore the relevance of its protein complexes in maintaining intestinal barrier function mediated by both autophagy and endocytic trafficking. For example, deficiency of Complex I component *Atg14* in mice triggers TNF-dependent villus atrophy and IEC apoptosis in the small intestine.^[240]^ Loss of *UVRAG* (a component of Complex II) in *D. melanogaster* ISCs disrupts enterocyte differentiation and endocytic trafficking, resulting in hyperproliferation, intestinal dysplasia and early death.^[241]^ Similarly, PI3KC3/Vps34 (in both Complex I and II) deficiency in zebrafish impairs PtdIns3P formation and junctional protein localization, causing intestinal epithelial injury and premature death.^[242]^ Altogether, this evidence illustrates BECN1’s role as a central hub linking immune regulation, stress adaptation, and dynamic membrane trafficking to maintain gut homeostasis.

### BECN1 in intestinal disease

Dysregulation of BECN1 has been implicated in human intestinal pathologies, including IBD, celiac disease, and colorectal cancer, where epithelial barrier dysfunction plays a central role in disease development and progression.^[243–248]^ In ulcerative colitis, BECN1 expression has been reported to increase in inflamed colonic mucosa relative to unaffected regions within the same patient and compared to individuals with non-inflammatory conditions such as irritable bowel syndrome^[248]^ (Table 1). This upregulation may represent a compensatory response aimed at counteracting inflammation-induced epithelial stress, including ER stress, oxidative stress, and DNA injury.^[45,236]^

Altered BECN1 levels are also associated with colorectal cancer. High expression correlates with longer overall survival,^[249]^ whereas reduced expression is linked to tumor progression, metastasis, and poorer prognosis^[246,250]^ (Table 1). While often attributed to impaired autophagy and apoptosis, emerging data highlight a role in suppressing epithelial-mesenchymal transition (EMT). In breast cancer models, loss of *Becn1* or its binding partner *Uvrag* led to CDH1 mislocalization, reducing membrane localization and increasing cytoplasmic accumulation, thereby promoting EMT and invasiveness.^[251]^ Given BECN1’s established role in regulating CDH1 trafficking in intestinal epithelial cells,^[47–49]^ it is plausible that reduced BECN1 expression compromises epithelial barrier integrity, facilitating both EMT and local tissue invasion in colorectal cancer. Thus, barrier dysfunction arising from defective BECN1-dependent trafficking may represent an early and mechanistically significant step in tumor initiation and progression, warranting further study.

Additional studies have identified BECN1 downregulation in other intestinal barrier disorders. In pediatric celiac disease, BECN1 expression is significantly reduced in intestinal biopsies from patients with active disease, although the downstream consequences remain unclear^[247]^ (Table 1). Similarly, in a rat model of irritable bowel syndrome, decreased colonic BECN1 levels were accompanied by increased inflammation, oxidative stress, and diminished MAP1LC3B (LC3-II), indicative of impaired autophagy flux^[252]^ (Table 1). Although mechanistic insights were not explored, these observations suggest a broader association between BECN1 insufficiency, stress responses, and compromised barrier integrity.

Altogether, evidence from both clinical and experimental studies illustrates BECN1’s role as a central hub linking immune regulation, stress adaptation, and dynamic membrane trafficking to maintain gut homeostasis and epithelial barrier function. Further investigation in larger, well-characterized patient cohorts, including those with IBD, celiac disease, colorectal cancer, and irritable bowel syndrome, may provide deeper insight into its context-specific functions and barrier-related disease relevance.

## BECN1 in the skin epithelium

### BECN1 in skin barrier homeostasis

Beyond the intestinal tract, the skin epithelium represents another barrier surface where BECN1 regulates its structural and functional integrity. As the body’s largest organ, the skin spans up to 2 m^2^ and forms the first line of defense against environmental insults such as pathogens, ultraviolet radiation, toxic chemicals, allergens, and mechanical injury.^[253]^ The epidermis, composed primarily of keratinocytes, forms a stratified squamous epithelium that undergoes continuous renewal and terminal differentiation. During this process, keratinocytes progressively lose their organelles and nuclei, and differentiate into corneocytes, forming the outermost cornified envelope, which is critical for barrier function.^[253]^

As the skin is a nutrient-poor microenvironment, autophagy is essential to recycle limited intracellular resources and maintain homeostasis.^[254,255]^ Under steady-state conditions, however, core autophagy genes appear largely dispensable for barrier structure. For example, epidermis-specific deletion of ATG7, ATG5 or ATG14 in mice results in normal skin morphology, cornification, and transepidermal water loss under baseline conditions, indicating that autophagy is largely dispensable for epidermal barrier development in the absence of stress.^[50,254,256]^ Autophagy-deficient models show that while basal autophagy does support keratinocyte viability and differentiation, it is not essential for barrier formation. For instance, siRNA-mediated knockdown of *ATG5* in human HaCaT keratinocytes inhibits both proliferation and differentiation following hydrogen sulfide-induced stimulation.^[257]^ Similarly, *BECN1* knockdown in organotypic epidermis delayed differentiation due to defects in intracellular digestion of proteins and organelles.^[258]^

However, under physiological stress or injury, autophagy becomes critical for epidermal repair and resilience. Keratinocytes lacking *Atg7* accumulate DNA damage, exhibit lipid dysregulation and undergo cell cycle arrest under oxidative stress, leading to impaired stress responses and accelerated aging. Similarly, mice deficient for *Atg5* or *Atg7* in keratinocytes display impaired re-epithelialization and fibroblast activation during wound healing.^[259]^ Thus, while autophagy is not essential for steady-state skin barrier structure and function, it is necessary for epidermal repair and regeneration under stress.

In contrast, BECN1 plays a uniquely indispensable role in epidermal homeostasis even under basal conditions. Keratinocyte-specific deletion of *Becn1* causes profound barrier dysfunction as neonatal mice lacking BECN1 exhibit taut, shiny skin, and die within 24 hours of birth due to rapid dehydration and excessive transepidermal water loss.^[50]^ These mice also exhibit weight loss, dehydration, and increased skin permeability, and severe defects in epidermal stratification and differentiation, phenotypes not observed in *Atg5-*, *Atg7-*, or *Atg14*-deficient mice. Mechanistically, BECN1 loss results in the accumulation of ITGA6 (integrin subunit alpha 6) in receptor-positive recycling endosomes that fail to colocalize with the early endosomal marker EEA1, indicating impaired early and recycling endosome function^[50]^ (Table 1). Notably, this endosomal stalling phenotype mirrors the cargo trafficking defects observed in IECs following *Becn1* deletion,^[47,49]^ underscoring a conserved role for BECN1 in regulating endocytic membrane trafficking across barrier tissues. While trafficking of junctional proteins such as CDH1 and OCLN was not directly investigated in skin, these findings highlight a broader role for BECN1 in regulating membrane trafficking pathways critical to skin barrier integrity.

The severe phenotypes observed in epidermal-specific *Becn1* knockout mice closely resemble those in DSG1 knockout mice, where loss of desmosomal cadherins disrupts the epidermal barrier and results in post-natal lethality.^[50,260,261]^ Retromer-mediated recycling of DSG1 is essential for keratinocyte differentiation and stratification.^[131]^ BECN1 regulates retromer recruitment and trafficking of surface receptors, such as CD36 (CD36 molecule), TREM2 (triggering receptor expressed on myeloid cells 2), and the Wnt receptor WLS/mig-14 (Wnt ligand secretion mediator/wntless homolog) in mammalian and *C. elegans* systems,^[235,262]^ suggesting BECN1-mediated endocytic trafficking is crucial for delivery and recycling of proteins required for epidermal development. This trafficking function, not shared by other core autophagy proteins, may explain the uniquely severe phenotype seen in *Becn1*-deficient skin models and highlights its broader importance across epithelial tissues.

### BECN1 in skin disease

Dysregulated BECN1 expression has also been implicated in human skin pathologies. In melanoma, SIRT1 (sirtuin 1)-mediated deacetylation of BECN1 promotes autophagic degradation of CDH1, enhancing EMT and invasive behavior. Pharmacological inhibition of autophagy with chloroquine restores CDH1 levels and reduces migration, suggesting a role for BECN1-regulated autophagy in junctional remodeling during tumor progression^[142]^ (Table 1). This illustrates how BECN1-dependent degradation of junctional proteins can compromise epithelial cohesion and polarity, suggesting that barrier-regulatory mechanisms may be co-opted in cancer to facilitate EMT and metastasis.

In chronic skin conditions, BECN1 levels are elevated in psoriatic lesions and keloid tissues, reflecting increased autophagic activity in hyperproliferative or fibrotic environments^[263,264]^ (Table 1). Although the precise mechanisms remain unclear, BECN1-mediated autophagy has been linked to both increased keratinocyte proliferation and the production of pro-inflammatory cytokines in these conditions.^[264,265]^ Conversely, hypertrophic scar tissues, which display distinct fibrotic and apoptotic profiles compared to keloids, display reduced autophagic activity, as evidenced by reduced MAP1LC3B and BECN1 levels^[266,267]^ (Table 1). This autophagy deficiency contributes to excessive fibroblast proliferation and extracellular matrix deposition that drives hypertrophic scar formation.^[266,267]^ Supporting this, treatment of hypertrophic scar fibroblasts with ursolic acid (an inducer of autophagy and apoptosis *via* BECN1 upregulation) reduced collagen synthesis and triggered apoptotic cell death.^[268]^

Although junctional protein trafficking was not examined in the studies above, defective ITGA6 trafficking and severe barrier disruption observed in *Becn1*-deficient skin,^[50]^ as discussed previously, suggest that impaired membrane trafficking may contribute to disease pathogenesis. This raises the possibility that altered BECN1 expression in skin disorders may affect not only autophagic and inflammatory pathways, but also junctional integrity and barrier function. Furthermore, these contrasting patterns underscore BECN1’s context-dependent roles in skin pathology, which may be influenced by its regulation of autophagy, membrane trafficking, and cell-cell adhesion.

Collectively, these findings highlight that while general autophagy contributes to keratinocyte differentiation and stress adaptation, BECN1 is uniquely required for skin barrier development. Through its capacity to coordinate endocytic trafficking and canonical autophagy, BECN1 orchestrates key processes in epithelial architecture and membrane protein recycling. Further research is required to explore whether BECN1 governs the trafficking of intercellular junctional proteins in the skin, which could clarify its distinct role in maintaining epidermal barrier integrity.

## Functions of BECN1 in endothelial barrier homeostasis

### BECN1 in endothelial homeostasis and stress adaptation

Endothelial barriers, such as the BBB, control selective permeability between the circulation and tissues. The BBB comprises tightly connected brain microvascular endothelial cells (BMECs), supported by astrocytes, pericytes, and perivascular microglia.^[269,270]^ While the roles of autophagy and endocytic trafficking in neuronal function are well established^[271–273]^, BECN1-mediated regulation of these pathways in endothelial barriers, particularly at the BBB, is now increasingly recognized as crucial for maintaining selective permeability and responding to stress.

Under physiological and mild stress conditions, BECN1-mediated autophagy preserves endothelial barrier integrity by regulating turnover of junctional proteins. For example, in low-serum conditions, autophagy regulates the localization of CLDN5 in BMECs to maintain barrier function.^[274]^ Under hypoxia, CAV1-mediated internalization of CLDN5 results in cytoplasmic accumulation, which is exacerbated in BECN1-deficient zebrafish. Restoring BECN1 alleviates this disruption by promoting autophagic clearance of CAV1-CLDN5 cytosolic aggregates to maintain endothelial junctional organization^[275]^ (Table 1). Consistent with this, autophagy-deficient mice exhibit cerebrovascular barrier dysfunction and reduced TJ protein expression, further supporting a protective role for BECN1 in endothelial homeostasis.^[276]^

#### Endocytic trafficking and retromer function

Beyond its role in autophagy, BECN1 also governs endocytic trafficking processes critical for endothelial health. In brain endothelial cells, disruption of the retromer complex impairs lysosomal clearance and weakens barrier integrity, suggesting that endocytic trafficking is essential for proteostasis and BBB maintenance.^[277]^ Although BECN1 was not directly tested in this context, its known role in recruiting and regulating the retromer complex points to a likely contribution to endothelial barrier stability.

#### Insights from neural cells and indirect barrier effects

Evidence from other neural cell types supports this broader function. In Purkinje neurons, BECN1 loss disrupts PtdIns3P generation and EEA1 recruitment to endosomes, resulting in enlarged LAMP1 (lysosomal associated membrane protein 1)-positive vesicles and aberrant cargo accumulation. This led to abnormal gait and ataxic behavior in mice^[51]^ (Table 1). BECN1 also regulates retromer-mediated recycling of the TGFB1/TGFbeta (transforming growth factor beta 1) receptor TGFBR1/ALK5 (transforming growth factor beta receptor 1), to RAB11A-positive endosomes, enabling neuroprotective signaling, whereas decreased BECN1, PIK3C3/Vps34, or UVRAG levels impair TGFB1 signaling and promote neuronal cell death^[278]^ (Table 1). Similarly, in astrocytes and microglia, BECN1 coordinates retromer recruitment to phagosome membranes for the recycling of phagocytic receptors such as CD36, TREM2 or SCARB1 (scavenger receptor class B member 1)^[262,279]^ (Table 1). Dysfunction in these glial pathways may indirectly compromise BBB integrity by impairing glial support functions. Together, these observations suggest that BECN1-mediated endocytic trafficking likely contributes both directly and indirectly to cerebrovascular barrier maintenance.

### BECN1 in endothelial barrier dysfunction and disease

#### BECN1 and traumatic brain injury (TBI)

Under pathological stress, BECN1-mediated autophagy can preserve or disrupt endothelial barrier integrity, depending on the nature and duration of cellular stress. In TBI, excessive autophagy is linked to BBB breakdown: elevated BECN1, MAP1LC3B (LC3-II), and SQSTM1/p62 (sequestosome 1) levels coincide with CLDN5 degradation and increased paracellular permeability^[280–282]^ (Table 1). Inhibiting autophagy with 3-Methyladenine or restoring PLA2G6/iPLA2beta (phospholipase A2 group VI) activity, a calcium-independent phospholipase A2 involved in membrane lipid homeostasis and stress signaling, reduces BECN1 expression, stabilizes TJ proteins and restores barrier integrity in bEnd.3 cells and *in vivo*^[281]^ (Table 1). Similarly, pharmacological inhibition of autophagy with DI-3n-butylphthalide improves endothelial junctional stability and upregulates TJ components such as OCLN in TBI models and SH-SY5Y/human BMECs. This protective effect is lost when DI-3n-butylphthalide is combined with rapamycin, reinforcing that excessive BECN1 activity and autophagy can undermine endothelial barrier integrity^[283]^ (Table 1).

#### BECN1-mediated autophagy in neurodegeneration

Autophagy-related modulation of BBB permeability, *via* BECN1, has also been implicated in neurodegenerative conditions. In Alzheimer’s disease, amyloid-β (Aβ1–42) oligomers induce BECN1-dependent autophagy in BMECs, leading to decreased ZO-1, OCLN, and CLDN5 levels. Blocking autophagy with 3-Methyladenine or siRNAs against MAP1LC3B restores junctional integrity, whereas rapamycin exacerbates barrier loss.^[284]^

#### Context-dependent roles in ischemic stroke

In acute ischemic stroke, BECN1’s role in the BBB is highly context dependent. In transient middle cerebral artery occlusion, ischemia-induced BBB disruption is linked to increased binding of RUBCN, a negative regulator of BECN1. In bEnd.3 cells subjected to oxygen-glucose deprivation, RUBCN expression increases, autophagic flux is impaired, and endothelial cell viability is reduced. Knockdown of RUBCN or disruption of its interaction with BECN1 restores autophagy, improves endothelial survival, and preserves BBB integrity. These findings suggest that targeting the RUBCN – BECN1 axis may hold therapeutic potential for modulating autophagy and protecting the BBB during ischemic injury^[285]^ (Table 1).

In contrast to transient ischemia, a lethal ischemia model in human umbilical vein endothelial cells shows suppression of canonical BECN1-dependent autophagy despite upregulation of other autophagy and lysosomal proteins, including ATG2A, ATG5, ATG7, LAMP1/2, CTSB (cathepsin B) and CTSD (cathepsin D).^[286]^ Functional studies confirm that canonical BECN1-dependent autophagy is not induced and that neither rapamycin nor chloroquine improves cell viability, suggesting that severe ischemia suppresses the canonical pathway and may instead shift cells toward lysosomal or non-canonical degradation mechanisms^[286]^ (Table 1). By contrast, under sublethal simulated ischemia/reperfusion conditions, BECN1 expression is upregulated in an NF-kappaB-dependent manner, accompanied by increased autophagic flux.^[287]^ Pharmacological inhibition of NF-kappaB suppresses autophagosome formation and improves endothelial viability, whereas rapamycin exacerbates injury^[287]^ (Table 1). These findings align with cardiac ischemia/reperfusion models, where reactive oxygen species production activates RELA/p65 (RELA proto-oncogene, NF-kB subunit), to drive BECN1 expression and excessive autophagy, an effect mitigated by NF-kappaB inhibition, which reduces BECN1 levels and limits myocardial damage^[288]^ (Table 1). Collectively, these findings highlight a redox-sensitive NF-kappaB-BECN1-axis activated under reperfusion stress that contributes to autophagic injury.

Together, these findings underscore the dualistic role for BECN1 in endothelial responses to ischemia where prolonged, severe deprivation suppresses canonical autophagy, whereas reperfusion stress activates NF-kappaB-driven BECN1 expression that promotes autophagic cell death. The balance of injury *versus* protection depends on the ischemic burden, lysosomal clearance capacity, and inflammatory milieu.

#### Endocytic trafficking dysfunction and barrier integrity

Dysregulated endocytic trafficking also appears to compromise barrier integrity across multiple tissues. In excitotoxic and ischemic models, dying neurons accumulate clathrin-, EEA1- and LAMP1-positive vesicles, indicating impaired endolysosomal processing^[289–291]^ Although BECN1 expression was not directly assessed, these trafficking defects resemble those observed in BECN1-deficient IECs, which exhibit enlarged endosomes, cargo retention, and defective lysosomal degradation.^[49,233]^

#### Inflammatory signaling and cytoskeletal dynamics in non-central nervous system (CNS) endothelium

In non-CNS endothelial tissues, BECN1 plays a significant role in inflammatory signaling and cytoskeletal dynamics that affect junctional integrity. For example, in pulmonary artery endothelial cells, BECN1 promotes RELA/p65 activation and CFL1 (cofilin1)-driven actin remodeling following thrombin challenge, impairing CDH5 junctional reassembly and exacerbating barrier dysfunction.^[53]^ BECN1 knockdown accelerates junctional recovery and restores barrier integrity.^[53]^ Similarly, in diabetic retinopathy, pharmacological downregulation of BECN1 by trimetazidine improves retinal endothelial barrier function under diabetic conditions.^[292]^

#### BECN1, RAB5 and CDH5 internalization

Although direct evidence in endothelial cells is limited, BECN1’s established role in RAB5-mediated endosomal sorting suggests it may regulate CDH5 internalization under inflammatory stress. Supporting this, in human pulmonary microvascular endothelial cells, lipopolysaccharide exposure increases RAB5 activity, promoting CDH5 endocytosis, loss of cell polarity, and barrier disruption. RAB5 depletion rescues these defects.^[293]^ Notably, similar trafficking defects are observed in BECN1-deficient intestinal epithelia, further reinforcing the idea that BECN1 helps preserve trafficking homeostasis and junctional stability across barrier tissues.^[49]^

Together, these findings highlight BECN1 as a multifaceted regulator of endothelial barrier function, with roles that are highly context dependent. Under normal physiological conditions, BECN1 maintains junctional homeostasis by coordinating autophagy and endocytic trafficking. In contrast, pathological stresses, such as ischemia-reperfusion injury, can shift this balance, with excessive or impaired BECN1 activity either promoting autophagic cell death or suppressing protective autophagy altogether. BECN1’s influence on cytoskeletal dynamics and junctional protein trafficking further shapes barrier integrity in diverse scenarios, including neurodegeneration, inflammation, and metabolic disease. While most studies have focused on its autophagy functions, emerging data underscore the importance of BECN1’s endocytic trafficking roles for sustaining junctional stability. Clarifying how these pathways intersect in tissue- and stress-specific contexts will be critical for realizing BECN1’s potential as a therapeutic target to preserve endothelial barrier function.

## Cross-tissue implications of BECN1 dysfunction in barrier-related comorbidities

### Role for BECN1 in shared barrier breakdown in chronic inflammatory diseases

Increasing evidence suggests that barrier dysfunction is not always tissue-restricted but can manifest systemically across organ systems. This is particularly evident in chronic inflammatory conditions such as psoriasis and IBD, where epidemiological and clinical data demonstrate a high degree of comorbidity.^[294–299]^ Individuals with psoriasis are significantly more likely to develop IBD, and *vice versa*, pointing to shared pathogenic mechanisms across epithelial and immune barriers. Intriguingly, therapeutic targeting of TNF in IBD has been associated with paradoxical induction or exacerbation of psoriatic lesions, further underscoring the interconnectedness of these diseases at both immunological and barrier levels.^[300,301]^ Although the molecular underpinnings of this cross-organ vulnerability remain to be fully defined, evidence from *Becn1*-deficient animal models supports the idea that BECN1 is a key node coordinating barrier function across tissues. Mice with impaired BECN1 activity exhibit concurrent epithelial defects in gut and skin, including compromised barrier integrity, disrupted epithelial differentiation, and aberrant immune cell infiltration.^[47–50,236]^ These findings suggest a systemic requirement for BECN1 to preserve homeostasis in epithelial tissues exposed to constant environmental stress.

### Shared BECN1-controlled pathways linking epithelial and immune dysfunction

Mechanistically, BECN1 orchestrates core processes that are foundational to barrier health, including autophagy, endocytic trafficking, cytokine secretion, and cell death that are fundamental to barrier integrity in multiple tissues. Dysregulation of these pathways may have widespread consequences beyond the tissue of origin. In the skin, for example, autophagy influences keratinocyte proliferation and inflammation,^[259,302–305]^ processes also central to intestinal epithelial function.^[45,47–49,107,221,224,227,229–231,236,240,306]^ Endocytic trafficking defects mediated by BECN1 deficiency may further impair the recycling of adhesion and junctional proteins,^[47–50]^ exacerbating barrier breakdown and inflammatory signaling across multiple sites. Moreover, transcriptomics analyses of psoriatic skin lesions and colon lesions from IBD patients reveal overlapping gene signatures between psoriasis and IBD, including perturbations in IL23A (interleukin 23 subunit alpha), TNF, and interferon pathways which intersect with autophagy-controlled networks regulated by BECN1.^[237,297,307–309]^ Such cross-tissue patterns reinforce the idea that BECN1 acts as a molecular integrator, coupling tissue-specific stress responses to systemic immune surveillance and inter-organ barrier crosstalk.

### Implications for comorbidity and future research

Taken together, these observations support a model where insufficient or dysregulated BECN1 may contribute to barrier failure in multiple epithelial organs, thereby predisposing individuals to risk of inflammatory comorbidities such as IBD and psoriasis. This is particularly relevant for patients with genetic susceptibilities or those receiving targeted biologics that may unmask latent barrier vulnerabilities. Therefore, understanding how BECN1 integrates epithelial and immune functions across tissues could inform the design of more holistic therapeutic approaches that stabilize barriers systemically, rather than addressing single organs in isolation.

## Therapeutic and clinical implications of BECN1 in barrier-associated diseases

### Balancing BECN1 activity: context matters

Emerging evidence across diverse organ systems highlights BECN1 as a promising therapeutic target for restoring barrier integrity and modulating inflammation in barrier-associated diseases. However, its pleiotropic roles in autophagy, endocytic trafficking, and immune regulation mean that both loss and overactivation of BECN1 can be detrimental. Hence, this balance must be finely tuned in a tissue- and context (e.g. stress)-specific manner. Devising strategies that target specific BECN1 functions will be difficult, though recent advances in the structural biology of Complex I/II (see below) will be useful in this context. Developing tissue-specific modulators of BECN1 (if required), however, may present a significant future challenge.

### Strategies to enhance BECN1 function in barrier loss

In the gastrointestinal tract, BECN1 deficiency leads to severe loss of epithelial architecture, mislocalized junctional proteins, and fatal barrier collapse. Therapeutic approaches that boost BECN1 function in this setting could restore autophagic flux and endocytic trafficking, thereby stabilizing epithelial junctions and preserving barrier homeostasis. One approach involves disrupting the inhibitory interaction between BECN1 and BCL2. In *Becn1*^*F121A*^ mice, which harbor a mutation that prevents BECN1-BCL2 binding, enhanced autophagy correlates with improved colonic mucus production and resistance to chemically induced colitis.^[236,310]^ These findings have inspired the development of small-molecule inhibitors of the BECN1-BCL2 complex, such as “Compound 35,” which has shown promise in pre-clinical models.^[311,312]^

Beyond small molecules, natural compounds and peptide-based therapies are under investigation. The dietary polyphenol piceatannol, for example, enhances BECN1 activity by modulating its phosphorylation at Ser295, offering a strategy to restore function without altering expression levels directly.^[313]^ Similarly, the Tat-BECLIN1 peptide has shown protective effects in models of infection, cardiac stress, and hepatic injury by selectively boosting BECN1-mediated autophagy.^[314]^ Given that BECN1 deficiency also compromises skin barrier integrity and promotes inflammation, such therapies may have broader applicability across epithelial tissues. Nonetheless, further studies are needed to assess the efficacy and safety of these interventions.

### Suppressing BECN1 activity when overactivation is harmful

Conversely, in certain CNS contexts, excessive BECN1-driven autophagy can compromise barrier function, as seen in ischemic or TBI models. Here, therapeutic suppression of BECN1 may help preserve endothelial tight junctions. For example, intranasal delivery of siRNA targeting *Becn1* using polyethyleneimine carriers has effectively reduced BECN1 expression in the prefrontal cortex of mice without eliciting immune activation or toxicity.^[315]^ This approach offers a noninvasive route to target BECN1 overactivation in neuroinflammatory and neurodegenerative disorders that disrupt the BBB.^[315]^

### Targeting BECN1’s endocytic trafficking function: an overlooked opportunity

Notably, most therapeutic strategies to date have focused on modulating BECN1-dependent autophagy, while largely overlooking its role in endocytic trafficking. Given accumulating evidence that BECN1 controls junctional stability *via* trafficking of adhesion molecules such as CDH1, OCLN, and CDH5, there is a strong rationale to develop strategies that selectively modulate BECN1’s trafficking functions. Thus, selective modulation of BECN1 complexes, such as promoting VPS34–UVRAG activity or inhibiting RUBCN-mediated suppression, could allow for more precise restoration of barrier integrity across tissues without triggering deleterious autophagic overactivation. Encouragingly, high-resolution structural insights into BECN1-VPS34 complexes now offer the prospect of structure-guided design of small molecules that differentially modulate Complex I (autophagy) and Complex II (endocytic trafficking) activities. These advances represent a crucial step toward the development of next-generation BECN1-targeted therapies tailored to specific barrier-related diseases.

## Concluding remarks

In conclusion, the evidence across multiple barrier tissues underscores BECN1’s pivotal role as an integrator of autophagy, endocytic trafficking, and stress adaptation. By maintaining junctional organization, regulating cargo recycling, and fine-tuning immune responses, BECN1 safeguards epithelial and endothelial integrity under both homeostatic and pathological conditions. While direct evidence remains limited in the context of barrier maintenance, BECN1 also has non-canonical roles, independent of autophagy and trafficking, that could be important. For example, the non-canonical role of BECN1 in cytokinetic abscission^[316,317]^ may be relevant in epithelial tissues, where continuous renewal and controlled cell shedding underpin barrier homeostasis. Hence, defects in cytokinesis could impair the orderly turnover of epithelial cells, leading to focal weaknesses in the barrier. Such additional functions, therefore, warrant further investigation. To this end, studies designed to formally dissect the relative contribution of each of BECN1’s functions will be valuable.

As research advances, carefully balancing BECN1’s multiple activities – harnessing its protective effects while preventing maladaptive overactivation – will be essential for developing next-generation therapies for barrier-related diseases. Achieving this precision could transform clinical approaches to inflammatory comorbidities that span the gut, skin, and brain, and unlock BECN1’s full therapeutic potential.

## Data Availability

Data sharing is not applicable to this article as no data were created or analyzed in this study.

## Abbreviations

AJ: adherens junction
BBB: blood-brain barrier
BMEC: brain microvascular endothelial cells
CNS: central nervous system
EMT: epithelial-mesenchymal transition
ERC: endocytic recycling component
GVB: gut-vascular barrier
IBD: inflammatory bowel disease
IEC: intestinal epithelial cells
ISC: intestinal stem cell
PtdIns3K: phosphatidylinositol 3-kinase
PtdIns3P: phosphatidylinositol 3-phosphate
TBI: traumatic brain injury
TJ: tight junction
